# The Gingiva from the Tissue Surrounding the Bone to the Tissue Regenerating the Bone: A Systematic Review of the Osteogenic Capacity of Gingival Mesenchymal Stem Cells in Preclinical Studies

**DOI:** 10.1155/2021/6698100

**Published:** 2021-06-12

**Authors:** Gamilah Al-Qadhi, Iman Aboushady, Niyaz Al-Sharabi

**Affiliations:** ^1^Department of Basic Dental Sciences, Faculty of Dentistry, University of Science and Technology, Yemen; ^2^Department of Oral Biology, Faculty of Dentistry, Cairo University, Cairo, Egypt; ^3^Department of Clinical Dentistry, Faculty of Medicine, University of Bergen, Bergen, Norway

## Abstract

The current review aims to systematically assess the osteogenic capacity of gingiva-derived mesenchymal stem cells (GMSCs) in preclinical studies. A comprehensive electronic search of PubMed, Embase, Web of Science, and Scopus databases, as well as a manual search of relevant references, was performed in June 2020 without date or language restrictions. Eligibility criteria were the following: studies that compared mesenchymal stem cells (MSCs) derived from the gingiva with other MSC sources (*in vitro* or *in vivo*) or cell-free scaffold (*in vivo*) and studies that reported at least one of the following outcomes: osteogenic potential and new bone formation for *in vitro* and *in vivo*, respectively. Moreover, the assessment of included studies was conducted using appropriate guidelines. From 646 initial retrieved studies, 35 full-text articles were subjected to further screening and 26 studies were selected (20 *in vitro* studies and 6 *in vivo* studies). GMSCs showed great proliferation capacity and expressed recognized mesenchymal stem cell markers, particularly CD90. *In vitro*, MSC sources including GMSCs were capable of undergoing osteogenic differentiation with less ability in GMSCs, while most *in vivo* studies confirmed the capacity of GMSCs to regenerate bony defects. Concerning the assessment of methodological quality, *in vitro* studies met the relevant guideline except in five areas: the sample size calculation, randomization, allocation concealment, implementation, and blinding, and *in vivo* publications had probably low risk of bias in most domains except in three areas: allocation concealment, attrition, and blinding items.

## 1. Introduction

From autogeneic grafts to NanoBone materials, various therapeutic strategies to promote bone regeneration have been established over the past years. Autogeneic, allogeneic, and xenogeneic bone grafts, different forms of natural or synthetic bone graft substitutes, bioactive molecule-augmented bone graft substitutes, cell-based bone graft substitutes, and NanoBone materials have been reported in preclinical and clinical fields [1].

However, their implantation in bone regeneration has been associated with different complications. Although autogeneic grafting is considered the gold standard therapy, donor site pain, extrasurgery demand, increased surgical time, and postoperative infection were reported in several studies as drawbacks. Similarly, allogeneic grafting combined with the absence of osteogenic potential, risk of viral contamination, and lack of biological and mechanical properties due to the irradiation process required for antimicrobial purposes was reported. Further, the disadvantages of xenografts were the lack of osteogenic ability and risk of immune rejection [2].

In addition to the lack of osteoinductive capability, slow degradation rate, and lack of mechanical properties, high pressure and temperature processing have been demonstrated in the bone graft substitutes, such as calcium salt-derived ceramics [3]. Augmentation of bone graft substitutes with growth factors such as bone morphogenetic protein results in accelerated bone healing [4]. It is clear that when materials are exposed to a biological environment, protein adsorption occurs immediately and subsequently mediates cell adhesion and proliferation processes. In comparison to conventional materials, nanomaterials may promote greater amounts of specific protein adsorption (e.g., albumin, laminin, collagen, fibronectin, and vitronectin). Not only the greater protein interactions but also the biomimetic size, larger surface area, and better mechanical properties make nanostructured material a good option to increase osteoblast functions (adhesion, proliferation, and differentiation) [5, 6]. Yet, the application of dental nanomaterial may induce cellular toxicity and need more investigations [7].

Consequently, these limitations have led researchers to improve the existing techniques or to develop new ones. Among the proposed promising strategy for bone regeneration is stem cell-based therapy, significantly mesenchymal stem cells (MSCs). These cells are considered major key players in transitional regenerative medicine [8], mainly due to their differentiation capacity into specific cell types, profuse secretion of soluble growth factors and cytokines, migration and homing potential to the site of injury, immunomodulatory effects on innate and adaptive immune cells, and anti-inflammatory effects [9].

Immunomodulatory and anti-inflammatory effects occur through cell-cell interactions or paracrine activities. Interferon-gamma (IFN*γ*) combined with Tumor Necrosis Factor (TNF) or Interleukin-1 (IL-1) stimulates MSCs to produce chemokine receptor ligands such as CXC-Chemokine Receptor 3 (CXCR3) ligand, which in turn recruit T cells resulting in the suppression of proliferation and activity of T cells in their local environment. Besides, MSCs mediate immunosuppression by secretion of secretome including cytokines, growth factors, anti-inflammatory factors, and exosomes that inhibit the proliferation and function of proinflammatory cells, such as T Helper 1 (TH1) and/or TH17 cells, macrophages, neutrophils, Natural Killer (NK) cells, and B cells, and stimulate anti-inflammatory cells, such as macrophages and regulatory T and B cells. Further, anti-inflammatory cells inhibit the function of proinflammatory cells and subsequently promote tissue healing [10].

MSCs can be used alone or in combination with a scaffold or carrier, which act as an extracellular template allowing stem cells to attach, proliferate, migrate, and differentiate into target cells. Various options in scaffold designing have been developed to direct and enhance osteogenic differentiation, for example, architecture modification (pore size, stiffness, and topography) [6], chemical stimulation through altering and adjusting the ratio of Hydroxyapatite (HA) to tricalcium phosphate (TCP) [11], biochemical stimulation by introduction of bioactive factors like dexamethasone or vitamin C [12], bone-specific growth factors [13], or addition of mineral fillers like Dicalcium Phosphate Dihydrate (DCPD) and hydraulic Calcium Silicate (CaSi) into Polylactic Acid (PLA) scaffolds [14]. More recently, a growing interest in 3D bioprinting techniques led to opening a new chapter for the production of the bioactive scaffold in bone regeneration [15].

Furthermore, mechanical stimulation can be used to enhance the proliferation and differentiation of MSCs into specific cell types [16]. In particular, the mechanical strain and biological fluid flow are converted into biochemical signals and then integrated into cellular responses through mechanotransduction. In bone tissue, osteocytes act as sensory cells responsible for this mechanotransduction, while osteoblasts and osteoclasts are the effector cells [17].

Several studies have been conducted using bone marrow mesenchymal stem cells (BMSCs) as a gold standard option for bone defects [18–20] and other dental MSC sources [21, 22]. Nevertheless, harvesting inadequate numbers of BMSCs, long expansion culture time, pain, and morbidity that may occur following the aspiration and collection processes still represent a major issue [23]. Therefore, alternative sources from other locations with a sufficient number of cells and easy isolation procedures have prospected. MSCs from oral tissues, including the dental pulp of permanent and deciduous teeth, periodontal ligaments, dental follicles, and dental papillae, were highly committed to differentiate into osteoblasts and bone cell precursors [24]. Furthermore, MSCs from diseased oral tissue, such as the human periapical cysts [14], inflamed dental pulp, and gingiva [25], provide a new tool for bone regeneration.

One of the attractive dental MSC sources is the gingiva. It is a part of the oral mucosal tissue, which surrounds and supports the teeth and alveolar bone and has essential roles ranging from acting as a mucosal barrier to participating in oral mucosal immunity. The gingiva also represents the biomedical waste that occurs because of common dental procedures such as tooth removal, gum or periodontal surgery, and dental implant surgery [26]. Recently, stem cells isolated from the gingiva captivate many researchers in the regenerative medicine field to investigate their features and ability to regenerate tissue, including bone [27].

A novel population of stem cells from gingival tissue called gingiva-derived mesenchymal stem cells (GMSCs) was isolated from humans and successfully verified by many researchers [28, 29]. Certainly, gingivae contain both neural crest-derived MSCs (90%) and mesoderm-derived MSCs (10%) with distinct stem cell properties. From clinical and laboratory aspects, the gingiva is considered an easily accessible convenient source with a less-invasive biopsy-taking technique and it is attainable to isolate sufficient numbers of MSCs from gingival tissue depending on their high proliferation rate [30].

In addition to their trilineage differentiation ability, self-renewal, and expression of mesenchymal surface markers, they exhibited immunomodulatory and anti-inflammatory properties that make them an attractive source for regenerative application [28]. These cells met the standard minimal criteria proposed by the International Society for Cellular Therapy [31]. Along with bone regeneration, previous studies have indicated that GMSCs had the capacity to regenerate the nerve [32, 33], muscle [34], tendon [35], skin [36], and cartilage and synovial tissues [37].

Equally important, the systematic reviews are ordinary procedures in clinical trials to assess the clinical efficiency of certain interventions while systematic reviews in the preclinical research community are still uncommon and need more attention to improve preclinical studies and in turn determine the first step in translating research from bench to patient [38].

Considering the above background, the current study is aimed at systematically reviewing the efficacy of applying GMSCs, as a newly introduced source, in bone regeneration in preclinical studies. This systematic review based on the following PICOS question: problem/population: bone defect in animal models (*in vivo*) and cell culture models (*in vitro*); intervention: GMSCs for both *in vitro* and *in vivo*; comparator or control: comparator: other sources (*in vitro*)/comparator or control: other sources and cell-free groups (*in vivo*); outcome: osteogenic capacity; and study design: preclinical studies. All items of PICOS have been used to formulate the following research question: will gingiva-derived mesenchymal stem cells be considered comparable or alternative to other MSC sources regarding bone regeneration in preclinical studies?

## 2. Materials and Methods

The current systematic review was conducted in accordance with the following guideline: Preferred Reporting Items for Systematic Reviews and Meta-Analyses (PRISMA) [39] (see supplementary material I for the PRISMA checklist).

### 2.1. Protocol Registration

The protocol of the current study was registered at the CAMARADES (Collaborative Approach to Meta-Analysis and Review of Animal Data from Experimental Studies) website at the following link: https://drive.google.com/file/d/1fkC_zkxGueWPyguYHAUH2SPLnJkwI3gK/view.

### 2.2. PICOS Question

The current systematic review was designed to answer the following question: will gingiva-derived mesenchymal stem cells be considered comparable or alternative to other MSC sources regarding bone regeneration in preclinical studies?

### 2.3. Eligibility Criteria

The eligibility criteria were established depending on the PICOS items. Therefore, any study that met the inclusion criteria was added as an eligible potential study. To be included, *in vivo* studies should consider the bone as a targeting area, and bone defects were induced in animals either by surgery or by disease regardless of species, age, and sex, as well as *in vitro* culture studies that compared MSCs derived from the gingiva and other MSC sources, *in vivo* studies that compared MSCs derived from the healthy gingiva and cell-free scaffold or other MSC sources, and studies that reported at least one of the following outcomes: new bone formation (*in vivo*) and osteogenic potential (*in vitro*).

On the other hand, publications that have the following criteria were excluded: targeted areas other than the bone, including the skin and gingiva, irrelevant intervention such as the inflamed gingiva, epithelial gingival cells or gingival fibroblasts, or induced pluripotent stem cells, as well as studies using derivatives of GMSCs such as Extracellular Vesicles (EVs), secretome, gene modification and cocultured cells, single-arm studies, reviews, opinion, and case studies.

### 2.4. Search Sources and Strategy

The popular electronic databases were systematically searched to identify the potential preclinical papers that assess the efficiency of GMSCs in regenerating the bone defects or osteodifferentiation *in vivo* and *in vitro*, respectively. The research was carried out in June 2020 and included the following databases: PubMed/MEDLINE, Embase, Web of Science, and Scopus, as well as a manual search of the reference lists of the relevant studies.

Based on “A step-by-step guide to systematically identify all relevant animal studies” [40], the research strategy was designed according to the type of the research engine using the following Search Component (SC) terms (medical subject headings (MeSH) terms, EMTREE, and free keywords) without date and language restrictions: SC1 (problem): (bone defect OR bone degradation OR bone disease OR bone disorder OR bone loss OR bone deformation OR bone destruction OR bone injury OR bone fracture) AND SC2 (intervention): (gingival mesenchymal stem cell OR gingival mesenchymal stem cells OR gingiva derived mesenchymal stem cell OR gingival tissue derived mesenchymal stem cells OR gingiva derived stromal cell OR gingiva derived stromal cells OR multipotent gingival stromal cell OR multipotent gingiva stromal cells OR multipotent gingiva progenitor cells OR gingiva stem cells) AND SC3 (control or compotator): (unloaded scaffold OR cell free scaffold) AND SC4 (outcome): (bone regeneration OR bone repair OR new bone formation OR bone healing OR bone tissue engineering OR bone remodeling OR osteogenesis OR osseointegration OR osteoconduction, osteogenic capacity (capability) OR osteogenic differentiation OR osteogenic potential) AND SC5 (study design): (animal model OR animal models OR experimental animal OR experimental animals OR laboratory animal OR laboratory animals OR *in vivo* study OR in vivo study OR *in vitro* study OR *in vitro* OR *in vitro* studies OR *in vitro* technique OR *in vitro* techniques OR cell culture technique OR cell culture method OR cell culture techniques OR culture technique OR culture techniques OR preclinical study) (see supplementary material II for the detailed search strategy).

### 2.5. Study Selection

All retrieved citations were imported and combined into the Mendeley folder, and the duplicated results were removed. Following that, two independent researchers (Al-Qadhi and Aboushady) did two phases of selection with specific exclusion items per phase. The first phase involved skimming the title and abstract of retrieved articles and then excluded the irrelevant studies, and the second phase involved scanning the full texts and excluded the irrelevant studies.

#### 2.5.1. Exclusion Criteria of Selection Phase I

- Review, case report, and expert opinion
- Irrelevant intervention, outcome, and problem
- Presence of any cofactors
- Single-arm studies

#### 2.5.2. Exclusion Criteria of Selection Phase II

- Review, case report, and expert opinion
- Irrelevant intervention, outcome, and problem
- Presence of any cofactors
- Absence of MSC source comparator
- Mixing cells into one culture
- Derivatives of a source of interest

In case of discrepancies, the two reviewers discussed the item and Al-Sharabi made the final decision. The reasons for exclusion were discussed and documented.

### 2.6. Data Extraction and Data Items

Data was collected and extracted using a preformed table designed by the authors. For *in vitro* culture studies, the following items were taken into consideration: study ID (author, year of publication), study design characteristics (experimental groups, source of stem cells, type of isolation method, type of culture medium, osteogenic induction period, density of cells/well, and type of scaffold or carrier if present), and outcome measures (proliferation potential, characterization of MSCs, and osteogenic differentiation).

Similarly, for *in vivo* studies, the following items were reported: study ID (author, year of publication), study design characteristics (experimental groups), animal model characteristics (species, gender, age, weight, and total numbers), intervention characteristics (source of stem cells, dose, mode of delivery, type of scaffold or carrier, and fate tracing), defect characteristics (site, size, way of induction, and time between induction and treatment), and outcome measures (characterization of MSCs, observation time points, method of analysis, and new bone formation).

### 2.7. Critical Appraisal of Individual Studies

To date, there is no specific risk-of-bias tool for quality assessment of *in vitro* culture experiments; therefore, we used the guidelines for reporting preclinical *in vitro* culture studies on dental materials based on the modification of the CONSORT checklist [41]. For *in vivo* studies, the risk of bias in included studies was evaluated using the Systematic Review Center for Laboratory Animal Experimentation (SYRCLE) risk-of-bias tool for animal intervention studies based on the Cochrane Collaboration RoB Tool with modification of some points [42]. The Health Assessment Workplace Collaborative (HAWC) online tool was used to manage the research process [43].

### 2.8. Summary Measures

To measure the primary outcome in *in vitro* culture studies, evidence of the formation of mineralized nodules was confirmed by Alizarin red staining or by the expression of osteogenic markers. Along the same line, outcome measures for *in vivo* studies were represented by the new bone formation that was assessed at least by histology, histochemistry, radiography, or gene expression of osteogenic markers.

### 2.9. Synthesis of Results

In the current systematic review, both outcome measures were performed in a qualitative description manner. The qualitative data synthesis was implanted because there was a marked variation in the way of presenting data among studies. Although most studies used the same outcome investigation method, some of them did not provide quantitative data; some of them used specific markers that differ from others and so on. Thus, the authors decided to illustrate the results in a qualitative manner rather than through a meta-analysis by trying the best to categorize data in a relevant chart.

## 3. Results

### 3.1. Study Selection

**Figure 1:**
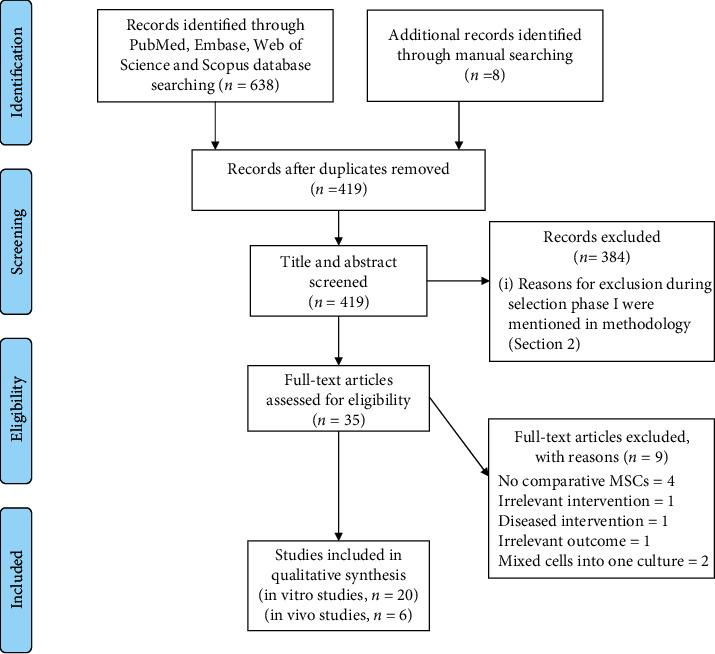
Flow diagram showing the different phases of literature screening for the systematic review process (editable file: PRISMA flow diagram, Liberati et al. 2009).

The followed search strategy resulted in the identification of a total of 646 possible relevant studies. Particularly, PubMed, Embase, Web of Science, and Scopus database searching provided 638 citations and manual searching identified additional 8 citations. Then, duplicated records were removed, resulting in 419 articles, and were subjected to skimming based on titles and abstracts. During this selection phase, 384 studies were excluded because they did not meet the criteria. Following that, 35 full-text remaining articles were selected for eligibility, and of these, 9 studies were excluded. The reason for exclusion was found in the supplementary material (see supplementary material III for the excluded studies with reasons). The previous steps are represented by the PRISMA flowchart (Figure 1).

### 3.2. Characteristics of *In Vitro* Included Studies

#### 3.2.1. Methods

A total of 26 studies were selected for the current systematic review. Of these, 20 *in vitro* were culture studies [44–63] and 6 were *in vivo* studies [64–69]. All papers were published in the English language, and the date of publication started in 2009. Regarding *in vitro* culture studies, most papers used humans as a source of MSCs except four studies that used animal sources such as mice [44, 45], pigs [46], and horses [47]. Concerning *in vivo* studies, all studies used humans as a source of MSCs except one study that used rabbits [48].

An enzymatic method of GMSC isolation was used in all *in vivo* and *in vitro* culture studies except in 2 *in vitro* culture studies where the outgrowth isolation method was used [49, 50]. In three *in vitro* studies, homogenous GMSCs were obtained after the isolation methods by colony-forming units [51] and fluorescence-activated cell sorting (FACS) [49], while a single-cell cloning method was used in only one *in vivo* study [52]. The osteogenic induction medium was used in all *in vitro* culture studies with an induction period that ranged from 14 to 28 days. The density of cells ranged from 2 × 10^3^ to 2 × 10^6^. Seven studies used scaffolds or carriers, commonly tricalcium phosphate (TCP) [49, 53–55] and alginate [56–58]. Six studies of 20 studies confirmed the culture results by an ectopic bone formation (Table 1).

#### 3.2.2. Type of Interventions

All studies compared GMSCs with dental or nondental MSC sources. In detail, studies compared GMSCs with periodontal ligament stem cells (PDLSCs) [59]; with dental pulp stem cells (DPSCs) [50, 54, 60]; with BMSCs [44, 49, 53, 55]; with buccal fat pad-derived mesenchymal stem cells (BFPMSCs) [53]; with PDLSCs and BMSCs [56, 57]; with PDLSCs and DPSCs [61]; with PDLSCs and dermal stem cells (DSCs) [55]; with PDLSCs and subcutaneous mesenchymal stem cells (ScMSCs) [47]; with BMSCs and submandibular salivary gland-derived mesenchymal stem cells (SSMSCs) [62]; with PDLSCs, DPSCs, and BMSCs [49]; with PDLSCs, BMSCs, adipose-derived stem cells (ADSCs), and periosteum-derived mesenchymal stem cells (PSCs) [46]; and with PDLSCs, DPSCs, DFSCs, BMSCs, ADSCs, and umbilical cord mesenchymal stem cells (UCMSCs) [57] (Table 2).

#### 3.2.3. Identification of Interventions

The proliferation rate of GMSCs was significantly higher in 10 studies compared to other MSC sources [44, 50, 51, 53, 55–57, 59, 62, 63]. However, they were proliferated effectively in the remaining studies. Note that 3 studies did not perform the proliferation assessment [58, 64, 65]. MSCs must be characterized by their morphological appearance and functionally identified by differentiating into adipocytes, chondroblasts, and osteoblasts as well as phenotypically by expressing MSC surface markers CD29, CD73, CD90, and CD105, with lacking expression of CD45, CD34, CD14 or CD11b, CD79 alpha or CD19, and human leukocyte antigen-antigen D-related (HLA-DR) surface molecules [31].

Clearly, 10 studies verified GMSCs by using three ways of ISCT identification and approved MSC characteristics [44, 46, 47, 49, 50, 53–55, 62, 63]. It is worth mentioning that some studies implemented three ways of ISCT, but two out of three differentiation assays were applied; for instance, GMSCs differentiated into osteoblasts and adipocytes in the following studies [59, 61, 65] and into osteoblasts but not adipocytes in one study [46]. Besides, some articles utilized one out of three required differentiation analyses and revealed that GMSCs differentiated into osteoblasts only [57, 58, 64]. Two studies did not perform multilineage functional verification [45, 60].

Most studies carried out immunophenotype analysis and confirmed that all MSC sources were positively expressed MSC markers with the percentage of >95% and were negative for hematopoietic markers with the percentage of <5%, excluding the two studies that did not provide sufficient CD marker analysis [57, 58], and one study did not establish the analysis [62] (Table 3).

#### 3.2.4. Primary Outcomes

To measure the primary outcome of *in vitro* culture studies, osteogenic differentiation was confirmed by the formation of mineralized nodules using a histological staining method, particularly Alizarin red staining, and/or by the expression of osteogenic markers. All *in vitro* culture studies confirmed that MSC sources including GMSCs were capable of undergoing osteogenic differentiation with different degrees. Obviously, eight articles reported that GMSCs had a lower degree of osteogenic potential than other MSC sources [47, 51, 56, 57, 60, 62, 63, 65].

However, similar numbers of articles stated that both GMSCs and other MSC sources formed calcified nodules without determining which one is stronger than the other [46, 49, 50, 53, 55, 58, 59, 64]. Only two studies have approved that GMSCs had a stronger ability to form mineralized nodules [44, 54]. One study reported that GMSCs had a moderate osteogenic capability particularly, lower than BMSCs and higher than SSMSCs [62], and another study showed that GMSCs had a similar osteogenic ability to PDLSCs, higher than DSCs [55].

Moreover, the majority of studies conducted additional confirmation methods by using stains such as alkaline phosphatase (ALP), Von Kossa, H&E, and immunochemical stains or by gene expression of osteogenic markers. All articles that used gene expression analysis clarified that MSC sources, including GMSCs, expressed the following markers: ALP, Runt-Related Transcription Factor 2 (Runx2), Osteonectin (OCN), COL1, and Osterix (OSX), and the results were in agreement with staining results (Table 3).

### 3.3. Characteristics of *In Vivo* Studies

#### 3.3.1. Methods

Due to limitations in the number of animal studies comparing GMSCs with other sources, the authors decided to include animal studies that compare GMSCs with the control groups as well. In addition to negative controls (scaffolds without stem cells) that were used in all *in vivo* studies, GMSCs were compared with BMSCs [48] and with PDLSCs and BMSCs [66]. Different animal species were used to create the bony defect, rabbits [48], mice [66–68], dogs [69], and rats [52]. The dose ranged from 1 × 10^6^ to 4 × 10^6^. Local application of MSCs was documented in four studies [48, 52, 66, 69], while two studies [67, 68] used a systemic delivery. Green fluorescent protein (GFP) was used as a fate tracer in four studies [52, 67–69], whereas PKH26 was used in one study [48], and no fate tracer was applied in one study [66]. Different locations for surgically created defects, including the tibia, maxilla, mandible, and calvaria with defect sizes that ranged from 0.5 mm to 6.0 mm, were used. Besides, various scaffolds include NanoBone [48], Modified Eagle's Medium (*α*-MEM) [67, 68], alginate [66], cell sheet [69], and type 1 collagen (COL1) gel [52]. There was no study that mentioned the time between induction and application of intervention, except one [69] (Table 4).

#### 3.3.2. Types of Interventions

All studies compared GMSCs with the cell-free scaffold. From 6 studies, one study compared GMSCs to BMSCs [48], and another study compared between GMSCs, BMSCs, and PDLSCs [66].

#### 3.3.3. Identification of Interventions

Four animal studies verified MSCs using three ways of ISCT characterization items. However, one paper affirmed one of three differentiation lineages [66], and one paper did not establish the multilineage differentiation analysis [48] (Table 5).

#### 3.3.4. Results of the Primary Outcome

The area of newly formed bone was detected in animal studies using Hematoxylin and Eosin (H&E) staining and/or Masson Trichrome (MT) or immunohistochemical staining. In terms of *in vivo* studies, more newly formed bone areas in GMSC groups were detected by H&E staining [48, 52, 67–69] with the exception of one article indicating that GMSCs had lower new bone formation in comparison to PDLSCs and BMSCs [66]. These results were validated by an abundance of red color indicating mature bone formation [48, 67, 68] and strong expression of GFP, Osteopontin (OPN), and COL1 [52] (Table 5).

### 3.4. Critical Appraisal within Sources of Evidence

All *in vitro* culture studies lacked particularly five items: the sample size calculation, sequence regeneration except one study which reported that randomization was done but without giving out how they did it [57], allocation concealment except three studies which implemented random slides for examination [47, 62, 63], implementation, and blinding. Concerning other items, most studies followed guidelines for reporting preclinical *in vitro* studies (see supplementary material IV for the detailed assessment).

**Figure 2:**
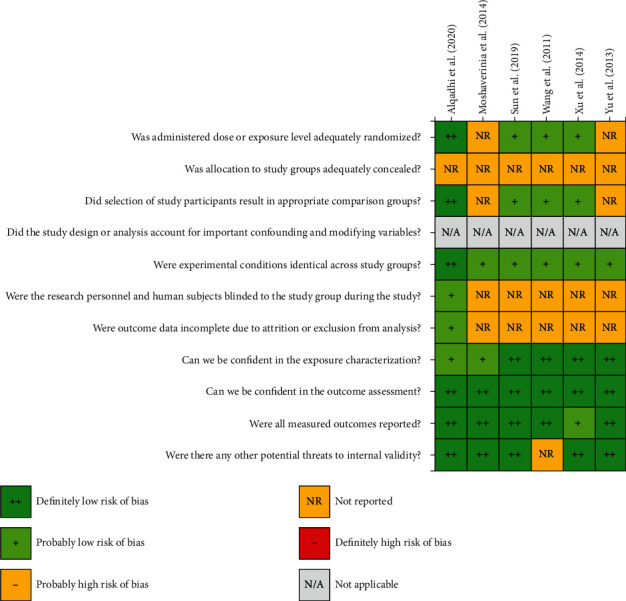
An assessment of the risk of bias among animal studies.

**Figure 3:**
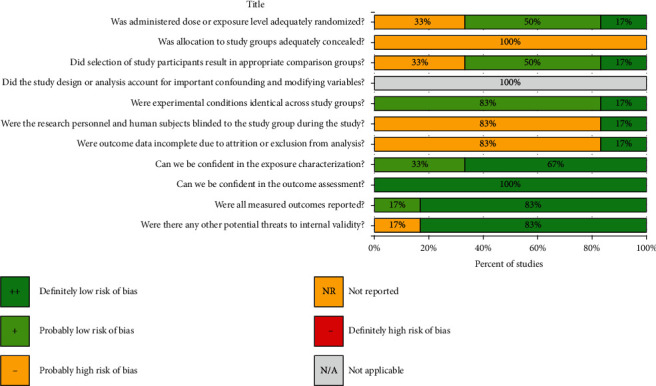
A study evaluation bar chart showing the percent of studies with each score, for each metric, in animal studies.

There are six domains to illustrate the risk of bias in animal studies. The selection domain results showed that not all *in vivo* studies performed allocation concealment. However, 4 of 6 studies performed randomization and provided similar baseline characteristics of experimental groups [48, 52, 67, 68]. The confounding domain was not applicable in the current systematic review as we excluded any studies that have confounding factors during the first phase of selection. The finding of the performance domain revealed that all studies showed identical experimental conditions among study groups. In contrast, only one study reported that independent researchers performed outcome analysis and gave details about the conditions of animals after the experiment [48]. Furthermore, all studies have a low risk of bias in detecting, reporting, and using appropriate statistical analysis domains. One paper did not use statistical analysis [52] (Figures 2 and 3).

### 3.5. Synthesis of Results

For the proliferation rate, the included studies showed four results: GMSCs had a significantly higher proliferation rate than other MSCs (*n* = 10), GMSCs had an effective proliferation rate somewhat similar to other MSCs (*n* = 7), GMSCs had a lower proliferation rate than other MSCs (*n* = 0), and the proliferation rate was unreported (*n* = 3). Regarding the characterization of interventions, the results of the included studies were identified based on three ways of identifications: morphological, functional, and phenotypical features. Data were presented as follows: definitely sufficient ways used to characterize the intervention (3 out 3) (*n* = 14*in vitro* and 4 *in vivo*), probably sufficient ways used to characterize the intervention (2 out 3) (*n* = 3*in vitro* and 2 *in vivo*), and insufficient ways used to characterize the intervention (1 out of 3) (*n* = 3*in vitro*).

**Figure 4:**
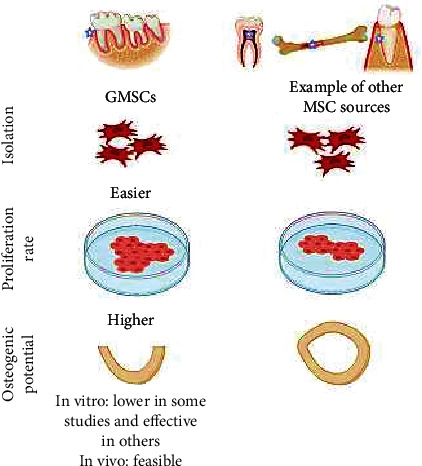
Schematic diagram represents the main finding of the current review.

The primary outcome of *in vitro* culture studies was described as follows: GMSCs had stronger osteogenic potential than other MSCs (*n* = 2), GMSCs had similar osteogenic potential to other MSCs (*n* = 8), and GMSCs had less osteogenic potential than other MSCs (*n* = 8). Along the same line, the *in vivo* outcome was presented as follows: more newly formed bone (*n* = 4), comparable newly formed bone (*n* = 1), and less newly formed bone (*n* = 1) (Figure 4).

## 4. Discussion

The main purpose of this systematic review was to summarize the role of gingiva-derived mesenchymal stem cells (GMSCs) and their effects on osteodifferentiation and bone regeneration both *in vitro* and *in vivo*. Overall, 26 studies were qualitatively reviewed. Twenty *in vitro* culture studies and six *in vivo* studies were mainly carried out to evaluate the proliferation and osteodifferentiation potential of GMSCs in comparison to other sources of MSCs or to nontreated GMSCs. The gingiva is recognized as a biological oral barrier against different insults and shows rapid regeneration without scar formation. This ability ascribes to the presence of an abundance of highly proliferative cells. These unique cell populations were firstly identified by Zhang and his colleagues as a cell population with similar stem cell-like properties [28].

Currently, these cells are commonly referred to as GMSCs and considered desirable stem cell sources because of their easy accessibility with limited morbidity. The multipotent differentiation capability of GMSCs has also been compared *in vivo* and *in vitro* with other stem cell types such as BMSCs, DPSCs, and PDLSCs. Therefore, the authors of this current review believe that in-depth assessment of the literature on preclinical *in vivo* and *in vitro* culture studies of GMSCs in bone tissue engineering and regenerative medicine is of great importance to explore the efficacy of GMSCs as a therapeutic source of MSCs.

The use of MSCs in clinical application generally depends on their biological properties including stemness and production of healing secretion factors. MSC is commonly isolated as a plastic-adherent cell population via either the enzymatic digestion method or the nonenzymatic digestion method. Researchers believe that cell isolation and expansion protocols influence stem cell quality, including yield, viability, and differentiation potential. Gingival tissues are commonly obtained after surgical removal of gingival tissue samples and either kept intact to grow out the adherent cells or digested by the use of specific enzymes to obtain single-cell suspensions.

Although no study to compare the two common isolation protocols for GMSCs is available to date, the majority of studies in this review display that GMSCs rapidly proliferate after both isolation methods, irrespective of their species. Likewise, in all studies evaluated in the present review, no study attempts to correlate this superior proliferative ability to any specific isolation method. On average, an increased number of cells with highly proliferative properties from the gingiva appeared after the enzymatic method. Among the studies evaluated in this systematic review (only three studies), a homogeneous population of stem cells was further selected using single-cell cloning [52], fluorescence-activated cell sorting [49], and colony-forming units [51].

Nevertheless, these data might hinder our precise conclusion of the advantages of isolation and purification methods on GMSC quality. In particular, since MSC quality is influenced by several factors during isolation and expansion producers, we recommend further assessment of GMSC isolation and expansion and establish a standard protocol before translating GMSCs to the clinical application. However, a review article by El-Sayed and Dörfer summarized various protocols of GMSC isolation and expansion and addressed some valuable information to achieve MSCs from gingival tissue [27].

To characterize GMSCs and compare their stemness properties to other types of stem cells *in vitro*, most of the current reviewed studies met the minimal criteria proposed by the International Society for Cellular Therapy for MSC characterization [31]. Self-renewal ability as a result of either asymmetrical or symmetrical cell division into different cells with distinct properties is considered the first basic cellular characteristic of stem cells. Almost all reviewed studies showed that GMSCs remain in a quiescent state after isolation and then propagate into a spindle-shaped morphology, similar to BMSCs, DPSCs, DPLSCs, ASCs, and others [46].

The morphological analysis of GMSCs showed no discernible difference between the reviewed studies as GMSCs displayed a stable phenotype in long-term culture and are nontumorigenic [58]. Likewise, GMSCs demonstrated a high ability for colony formation in two-dimensional (2D) culture and high viability rates in RGD-modified alginate hydrogel microspheres similar to BMSCs [54]. Admittedly, MSCs, as primary cells, show some *in vitro* proliferation potential and differentiation capability to a certain passage before they undergo considerable cellular alterations and senescence [70, 71]. On the contrary, GMSCs showed some remarkable growth characteristics in comparison to the other stem cell sources. In comparison to BMSCs, Tomar et al. reported that human gingival tissues generated highly proliferative, homogenous fibroblast spindle-like cells with a normal diploid number of chromosomes and normal karyotype even in the late passage [58].

Additionally, human GMSCs display highly growth properties than human BMSCs and PDLSCs, while a short doubling time of GMSCs was observed [62, 66]. GMSCs manifested higher proliferative rates than DPSCs at an early passage with more resistance to induced-oxidative stress and aging in long-term culture [61]. These significant properties are attributed to the continuous activation of the telomerase enzyme in GMSCs even in long-term cultures and to the heterogeneity of other types of stem cells [58].

On the other hand, GMSCs disclosed some unremarkable proliferative rates in comparison to other sources of stem cells, including human BMSCs [62] and pig BMSCs and PSCs [46]. Moreover, the low colony-forming unit-fibroblast (CFU-F) ability of GMSCs in comparison to human DSCs was reported [55]. The heterogeneity of the data showed in the current review of the literature can be attributed either to the donor- and tissue-dependent variations or to the cell isolation and expansion procedures. Substantial cell-to-cell variation among MSCs within a single population is thought to play a significant role in the experiments and outcomes [72]. Further studies are needed to investigate the effects of different culture conditions and properties of different GMSC subpopulations.

Nevertheless, the colony-forming assay with increased cell growth demonstrated that GMSCs exhibit a significantly higher proliferation potential than other stem cell sources. This result may be due to the biological function of the gingiva because it shows some exceptional reparative/regenerative potential after wounding. In terms of easy *in vitro* isolation with a large scale of cell expansion without significant phenotype and genotype alterations, GMSCs can be used as an attractive alternative source to BMSCs in stem cell research studies. Similar to standard characterizations of MSCs from other tissue sources, almost all the reviewed studies showed that GMSCs uniformly expressed certain stem cell markers, including CD90, CD105, CD146, and CD73 (all above 95%), and did not express hematopoietic stem cell markers CD45 and CD35. Even though the gingiva is densely vascular, these data might confirm the stromal origin of isolated cells without contamination with hematopoietic precursor cells.

However, this might not be necessarily correlated with the ability of MSCs to differentiate into other types of cells, including bone-forming cells. The majority of studies in the current review show the ability of GMSCs for *in vitro* osteodifferentiation under established osteoblast lineage-specific factors with remarkable changes in protein, gene, and miRNA levels. Unremarkable findings respective to GMSC osteodifferentiation potential in comparison to other stem cell types were also shown. GMSCs displayed less osteogenic potential than other MSC sources, while the high expression level of MSC surface markers was reported [47, 51, 56, 57, 62, 63, 65].

A study by Gao et al. demonstrated that human GMSCs with higher expression of CD90 in comparison to other stem cells of dental origin, DPSCs and PDLSCs, had a moderate osteogenic potential [55]. Two studies showed that GMSCs had better osteogenic capacity than BMSCs and DPSCs [44, 54]. Successful osteodifferentiation of GMSCs has also been shown in various studies and verified by staining of differentiated cells with Alizarin red or Von Kosssa and/or by expression of bone-related markers including Runx2, ALP, OCN, OPN, OSX, COL1, and COL3 [27]. GMSCs seeded in the 3D culture significantly increased the level of alkaline phosphatase in comparison to those from the 2D culture [73].

The heterogeneity of expression of several MSC surface markers is believed to arise from different subgroups within the MSC population, hence influencing significantly the MSC potency, including their osteodifferentiation potential [74]. In this regard, when correlating the osteogenic properties of GMSCs to the expression of MSC surface markers, in only one *in vitro* culture study, CD90 expression was evaluated during the osteodifferentiation of both BMSCs and GMSCs. Notably, the expression level of CD90 in BMSCs was gradually lost during their osteodifferentiation, while it stayed very high in GMSCs with a remarkable mineralized nodule formation [44]. CD90 antigen is one of the important markers of MSCs, and it can possibly serve as an index for CFU-F enrichment with strong osteogenic differentiation potential [75].

CD90 (+) subpopulation from ADSCs was characterized with higher osteogenic potential identified via alkaline phosphatase (ALP) and Alizarin red staining [76]. Although previous studies recommended STRO-1 for the enrichment of MSC-like subpopulation from the gingiva and other connective tissues [29], a low percentage of MSCs and reduced level of STRO-1 during culture expansion were reported [77]. Moreover, although such findings highlighted the significant roles of stem cell markers on selecting GMSCs with higher osteodifferentiation potential, further investigations on the influences of site and donor variations, experimental designs, and isolation methods are necessary.

To research the *in vivo* bone formation ability of GMSCs in different critical bone defects, in six *in vivo* studies, GMSCs were either injected systematically or mixed with different carriers before they were locally implanted. Although systematic delivery keeps cells in contact with oxygen and nutrient supply, some research studies approved that it may result in the concentration of MSCs in different sites of the body such as the lung, driven by inflamed organs. The benefit of local transplantation is the close proximity of MSCs to the site of the bone defect. However, the survival of MSCs may be affected due to insufficient oxygen and nutrients at the sites of injection. Therefore, further approaches should be applied to enhance cell engraftment and survival upon therapeutic transplantations [78].

In animal studies included in the current review, almost all GMSCs formed new bone tissues with a lamellate pattern yet in different degrees. Wang et al. demonstrated that GMSCs loaded on collagen scaffolds have more expression of OPN and type 1 collagen when they were implanted in critical-sized mandibular and calvarial defects in rats than the free-cell scaffold group. Moreover, the newly formed bone was detected in both models eight weeks postoperatively [52].

Similarly, GMSCs were implanted systematically via the tail vein into mandibular bone defects in mice; after three weeks of transplantation, new bone formation was significantly higher in the GMSC-loaded group than the unloaded one [68]. In a recent study, Al-Qadhi et al. also reported that GMSCs loaded on NanoBone were able to regenerate bone tissue in critical-sized bone defects in rabbits in a way comparable to BMSCs [48]. Admittedly, a desirable cell carrier should provide the biological template needed for cellular growth, differentiation, and tissue formation [79]. Despite the lower expression of bone-forming markers of GMSCs in comparison to that of BMSCs or PDLSCs, encapsulated GMSCs in RGD-modified alginate microspheres showed an ability to form new bone tissue after 8 weeks in a critical-sized calvarial defect in mice [66]. Ten weeks after implant recovery, GMSC- and BMSC-seeded biografts also showed a higher mineralized tissue with strong OCN expression than the unseeded one [58].

**Figure 5:**
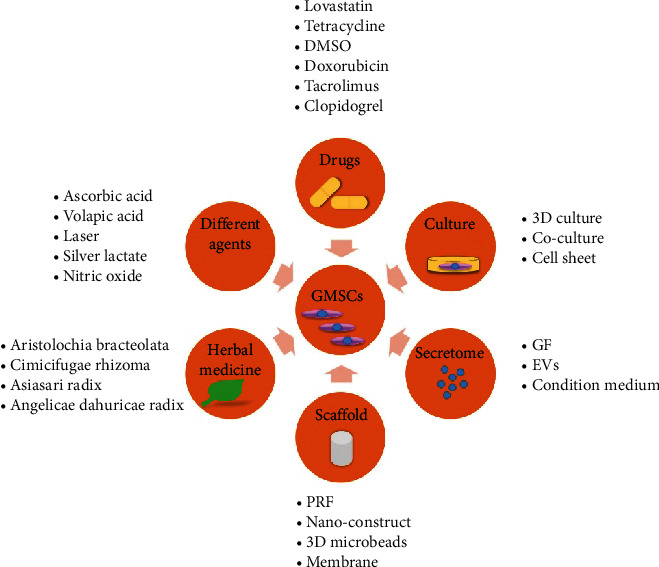
Different factors influence the osteogenic potential of GMSCs.

In particular, GMSCs mixed with other cells in one culture or one construct lead to enhanced osteogenic differentiation [80, 81]. It is therefore important to realize that many factors might influence the regenerative capacity of MSCs, including different culture conditions and cell carriers. Indeed, various articles have demonstrated the positive or negative impact of different factors on the viability and osteogenic potential of GMSCs. These factors include the following: conditioned medium [82], growth factors [83, 84], drugs [85, 86], herbal medicine [87], and scaffold type [88] (Figure 5). However, studies using exogenous factors or coculture systems were excluded from this systematic review to precisely conclude the osteodifferentiation potential of GMSCs without any synergistic or confounding factors.

## 5. Limitation in the Current Reviewed Studies

Even though *in vitro* culture preclinical research studies are considered a basic step for future research or for any novel therapeutic approach, it is worth mentioning that the methodological quality analysis of all evaluated *in vitro* culture studies shows some bias, such as lack of repetition of the experiments and lack of randomization, blinding, and sample size calculation. In spite of these factors that can influence the scientific validity of experimental results as all phases of the research process are interlinked, some researchers thought that those quality analyses could not be applied to *in vitro* culture studies [89]. For instance, the insufficient sample size might give incorrect results, which then may affect the outcomes of preclinical studies and in turn future clinical trials. We, therefore, found it is reasonable to apply the modified CONSORT guidelines [41] to *in vitro* culture studies to emphasize the importance of avoiding bias in future MSC research studies.

Importantly, no clinical trials of bone tissue engineering by GMSCs were found. The authors believe that the reasons might ascribe either to an early judgment of the outcomes from preclinical studies or to insufficient data collected. To confirm whether the outcomes of *in vivo* studies are not influenced by intended or unintended bias, all animal studies should have a clear study design. These parameters should include reporting of randomization, baseline characteristics (age, sex, and weight), animal housing conditions, blinding, animals enrolled and attrition, defect characteristics, follow-up, disclosing any adverse effects during and after the intervention, sample size, methods of size calculation, and reporting statistical analysis. This information will significantly improve the internal and external validity of any study and will also help researchers to publish a high level of scientific evidence similar to human RCTs.

With respect to the *in vivo* studies, most studies revealed a probably low risk of bias in sequence, detection, reporting, and one metric of performance (identical across group study) domains, while another metric of performance (blinding) and attrition domains were not reported.

## 6. Conclusion

Although GMSCs show similar or lower osteodifferentiation potential *in vitro* culture studies compared to other MSC sources, regenerating bony defects *in vivo* was significantly feasible with GMSCs. The easy accessibility and highly proliferative ability of GMSCs with a short doubling time could make them an attractive alternative source in the field of bone tissue engineering. However, the limited *in vitro* degree of osteodifferentiation potential of GMSCs remains a disadvantageous outcome. Therefore, further optimization of the *in vitro* culture conditions is needed.

In the same way, due to the insufficient number of *in vivo* studies that highlight the role of GMSCs in bone regeneration and based on the quality of the literature, using appropriate animal models with critical bone defects rather than ectopic models is highly important before going into a clinical application. Despite such findings for the benefit of using GMSCs in bone regeneration, well-designed preclinical studies that follow rigorous guidelines and manage a range of conditions such as experimental models, differentiating factors, culture media, and biological activity, cost-effectiveness, and safety of GMSCs are required.

## Figures and Tables

**Table 1:** Summary of findings for *in vitro* studies (methodology).

No.	Author and year of publication	Source of stem cells	Isolation method	Type of culture	Experimental groups			Osteogenic induction period (days)	Density of cells/well	Scaffold or carrier if present
					Stem cell-based intervention (source of interest)	Stem cell-based intervention (other sources)	Control group			
*In vitro* studies without confirmation of an ectopic bone formation										
1	Abedian et al. (2020) [59]	Human	Enzymatic	OIM	GMSCs	PDLSCs	Uninduced cells	14	12 × 10^3^	—
2	Kamel et al. (2019) [64]	Human	Enzymatic	OIM	GMSCs	DPSCs	Uninduced cells	21	2 × 10^5^	—
3	Sun et al. (2019) [44]	Mice	Enzymatic	OIM	GMSCs	BMSCs	Cell-free medium	21	5 × 10^4^	—
4	Xing et al. (2019) [61]	Human	Enzymatic^∗^	OIM	GMSCs	DPSCs PDLSCs	—	21	2 × 10^5^	—
5	Angelopoulos et al. (2018) [56]	Human	Outgrowth	OIM	GMSCs	DPSCs	Cell-free medium	28	7 × 10^5^	—
6	Zhang et al. (2018) [57]	Human	Enzymatic	OIM	GMSCs	DPSCs PDLSCs DFSCs BMSCs ADSCs UCMSCs	—	28	2.5 × 10^5^	—
7	Ghaderi et al. (2018) [53]	Human	Enzymatic	OIM	GMSCs	BFPMSCs	Uninduced cells	21	1 × 10^5^	—
8	Aboushady et al. (2018) [62]	Rats	Enzymatic	OIM	GMSCs	BMSCs SSMSCs	—	14	2 × 10^3^	—
9	Ansari et al. (2017) [54]	Human	Enzymatic	OIM	GMSCs	BMSCs	Cell-free scaffold	28	1 × 10^6^	Alginate-GelMA^∗^ hydrogel
10	Kaibuchi et al. (2017) [46]	Mini pig	Enzymatic	OIM	GMSCs	BMSCs ASCs PSCs PDLSCs	—	21	1 × 10^3^	—
11	Gao et al. (2014) [55]	Human	Enzymatic^∗∗^	OIM	GMSCs	PDLSCs DSCs	—	21	2 × 10^4^	—
12	Gay et al. (2014) [49]	Human	Enzymatic^∗^^#^	OIM	GMSCs	PDLSCs DPSCs BMSCs	Uninduced cells	28	5 × 10^3^	—
13	Yang et al. (2013) [60]	Human	Enzymatic	OIM	GMSCs	PDLSCs	Uninduced cells	28	1 × 10^5^	—
14	Moshaverinia et al. (2012) [45]	Human	Enzymatic	OIM	GMSCs	PDLSCs BMSCs	Cell-free scaffold	28	1 × 10^6^	3D alginate
*In vitro* studies confirmed the results by ectopic bone formation										
15	Zorin et al. (2014) [63]	Human	Enzymatic	OIM	GMSCs	BMSCs	—	14	3 × 10^3^	OCP ceramic granules B-TCP
16	Moshaverinia et al. (2013) [65]	Human	Enzymatic	OIM	GMSCs	PDLSCs BMSCs	Cell-free scaffold	28	0.5‐1 × 10^6^	3D alginate microbeads
17	Otabe et al. (2012) [50]	Human	Enzymatic	OIM	GMSCs	DPSCs	—	21	2.5 × 10^5^	B-TCP
18	Mensing et al. (2011) [47]	Horse	Enzymatic	OIM	GMSCs	PDLSCs ScMSCs	Uninduced cells	35	0.2 × 10^5^	—
19	Fournier et al. (2010) [51]	Human	Outgrowth^#^	OIM	Induced GMPC	GMPC (*in vitro*) BMSCs (ectopic)	Gingival fibroblast (*in vitro*) Cell-free carrier (ectopic)	21	2 × 10^6^	HA carrier
20	Tomar et al. (2010) [58]	Human	Enzymatic^##^	OIM	GMSCs	BMSCs (ectopic)	Cell-free scaffold (ectopic)	21-25 (*in vitro*) 10 (ectopic)	2‐5 × 10^3^ (*in vitro*) 1 × 10^6^ (ectopic)	HA/TCP

^∗^The outgrowth method instead of the digestion method to obtain cells from PDL. ^∗∗^Isolated cells were further selected via the single-cell cloning method. ^∗^^#^Homogenous population of cells was obtained by fluorescence-activated cell sorting after the enzymatic isolation method. ^#^GMSCs were obtained by the outgrowth method followed by colony-forming units. ^##^GMSCs were obtained by two times the enzymatic method, while BMSCs were obtained by density gradient centrifugation using Ficoll-Hypaque.

**Table 2:** Summary of findings for *in vitro* studies (identification of MSC results).

No.	Author and year of publication	Proliferation potential		Morphological appearance		Functional verification (trilineage differentiation)		Phenotypic verification (CD markers)	
		GMSCs	Other sources	GMSCs	Other sources	GMSCs	Other sources	GMSCs	Other sources
1	Abedian et al. (2020) [59]	Significantly higher	Effective	Fibroblast spindle-like cells	Fibroblast spindle-like cells	Osteogenic Adipogenic	Osteogenic Adipogenic	✓: CD105, CD90, CD73 ✖: CD34, CD45	✓: CD105, CD90, CD73 ✖: CD34, CD45
2	Kamel et al. (2019) [64]	Effective	Significantly higher	Fibroblast spindle-like cells	Fibroblast spindle-like cells	ND	ND	✓: CD105, CD90 ✖: CD45	✓: CD105, CD90 ✖: CD45^∗^ Data not reported
3	Sun et al. (2019) [44]	Significantly higher	Effective	Fibroblast spindle-like cells	Fibroblast spindle-like cells	Osteogenic Adipogenic Chondrogenic	Osteogenic Adipogenic Chondrogenic	✓: CD105, CD90 ✖: CD45, CD19	✓: CD105, CD90 ✖: CD45, CD19
4	Xing et al. (2019) [61]	Significantly higher	Effective	Fibroblast spiral-like cells	Fibroblast spiral-like cells	Osteogenic Adipogenic Chondrogenic	Osteogenic Adipogenic Chondrogenic	✓: CD105, CD90, CD73, CD44 ✖: CD34, CD11b, CD19, CD45, HLA-DR	✓: CD105, CD90, CD73, CD44 ✖: CD34, CD11b, CD19, CD45, HLA-DR
5	Angelopoulos et al. (2018) [56]	Significantly higher	Effective	Fibroblast spindle-like cells	Fibroblast spindle-like cells	Osteogenic Adipogenic Chondrogenic Angiogenic	Osteogenic Adipogenic Chondrogenic Angiogenic	✓: CD105, CD90, CD73, CD44 ✖: CD34, CD45, CD11b, HLA-DR	✓: CD105, CD90, CD73, CD44 ✖: CD34, CD45, CD11b, HLA-DR
6	Zhang et al. (2018) [57]	Comparably effective	Significantly higher in 4 dental MSCs and UCMSCs than BMSCs & ADSCs	Fibroblast-like cells	Fibroblast-like cells	Osteogenic Adipogenic Chondrogenic	Osteogenic Adipogenic Chondrogenic	✓: CD105, CD90, CD73, CD44 ✖: CD34, CD11b, CD19, CD45, HLA-DR	✓: CD105, CD90, CD73, CD44 ✖: CD34, CD11b, CD19, CD45, HLA-DR
7	Ghaderi et al. (2018) [53]	ND	ND	ND	ND	Osteogenic Adipogenic	Osteogenic Adipogenic	✓: CD166, CD105, CD90, CD73, CD44 ✖: CD14, CD34, CD45	✓: CD166, CD105, CD90, CD73, CD44 ✖: CD14, CD34, CD45
8	Aboushady et al. (2018) [62]	Moderately effective (less than BMSCs and more than SSMSCs)	Significantly higher in BMSCs	Fibroblast spindle-like cells	Fibroblast spindle-like cells	ND	ND	ND	ND
9	Ansari et al. (2017) [54]	ND	ND	ND	ND	Osteogenic	Osteogenic	✓: CD146, STRO-1	✓: CD146, STRO-1
10	Kaibuchi et al. (2017) [46]	Highest proliferation	Higher proliferation in BMSCs and PDLSCs followed by PSCs and ADSCs	ND	ND	Osteogenic NO: adipogenic	Osteogenic Adipogenic	✓: CD29, CD44, CD90 ✖: CD31	✓: CD29, CD44, CD90 ✖: CD31
11	Gao et al. (2014) [55]	Significantly higher than PDLSCs but lower than DSCs	Effective	Fibroblast spindle-like cells	Fibroblast spindle-like cells	Osteogenic Adipogenic Odontogenic^∗^	Osteogenic Adipogenic	✓: CD146, CD105, CD90, CD29, STRO-1 ✖: CD34, CD45	✓: CD146, CD105, CD90, CD29, STRO-1 ✖: CD34, CD45
12	Gay et al. (2014) [49]	ND	ND	Spindle-like cells	Spindle-like cells appear in immunoflouresence pictures	Osteogenic	Osteogenic	✓: CD105, CD29, STRO-1, integrin b1, SSEA4, OCT4	✓: CD105, CD29, STRO-1, integrin b1, SSEA4, OCT4
13	Yang et al. (2013) [60]	Significantly higher	Effective	Fibroblast spindle-like cells	Fibroblast spindle-like cells	Osteogenic Adipogenic Chondrogenic	Osteogenic Adipogenic Chondrogenic	✓: CD146, CD105, CD90, CD29, STRO-1 ✖: CD31, CD34	✓: CD146, CD105, CD90, CD29, STRO-1 ✖: CD31, CD34
14	Moshaverinia et al. (2012) [45]	Significantly higher in comparison to BMSCs	Significantly higher in PDLSCs than BMSCs	ND	ND	Osteogenic Adipogenic	Osteogenic Adipogenic	✓: CD146	✓: CD146
15	Zorin et al. (2014) [63]	Significantly higher	Effective	Fibroblast spindle-like cells	Fibroblast spindle-like cells	Osteogenic Adipogenic Chondrogenic	Osteogenic Adipogenic Chondrogenic	✓: CD146, CD105, CD90, CD73 ✖: CD34, CD45	✓: CD146, CD105, CD90, CD73 ✖: CD34, CD45
16	Moshaverinia et al. (2013) [65]	Significantly higher	Significantly higher (PDLSCs) Effective (BMSCs)	ND	ND	Osteogenic	Osteogenic	✓: CD146, CD73 ✖: CD34	✓: CD146, CD73 ✖: CD34
17	Otabe et al. (2012) [50]	Effective	Effective	ND	ND	Osteogenic Adipogenic Chondrogenic	Osteogenic Adipogenic Chondrogenic	✓: CD166, CD146, CD105, CD90, CD44 ✖: CD34, CD45, CD117	✓: CD166, CD146, CD105, CD90, CD44 ✖: CD34, CD45, CD117
18	Mensing et al. (2011) [47]	Effective	Significantly higher in ScMSCs followed by PDLSCs	Fibroblast spindle-like cells	Fibroblast spindle-like cells	Osteogenic Adipogenic Chondrogenic	Osteogenic Adipogenic Chondrogenic	✓: CD105, CD90 ✖: CD31	✓: CD105, CD90 ✖: CD31
19	Fournier et al. (2010) [51]	NS (not specified)	NS	Fibroblast-like cells	Fibroblast-like cells	Osteogenic Adipogenic Chondrogenic	ND	✓: CD146, CD105, CD90, CD73, CD44, CD29, STRO-1 ✖: CD34, CD45, CD117, CD200, HLA-DR	✓: CD105, CD90, CD73, CD44, CD29 ✖: CD34, CD45, CD117, CD200, HLA-DR
20	Tomar et al. (2010) [58]	Significantly higher	Effective	Fibroblast spindle-like cells	Fibroblast spindle-like cells	Osteogenic Adipogenic Chondrogenic	ND	✓: CD105, CD90, CD73, CD44, CD29, HLA-ABC ✖: CD14, CD34, CD45, HLA-DR (more stable)	✓: CD105, CD90, CD73, CD44, CD29 ✖: CD14, CD34, CD45, HLA-DR

**Table 3:** Summary of findings for *in vitro* studies (primary outcome: osteogenic potential).

No.	Author and year of publication	Tissue culture staining				Gene expression of osteogenic markers		
		Type of stain	GMSCs	Other sources	Control	GMSCs	Other sources	Control
*In vitro* studies without confirmation of an ectopic bone formation								
1	Abedian et al. (2020) [59]	Alizarin red staining	Formation of mineralized nodules	Similar formation of mineralized nodules	None	Expressed: ALP (24.2 fold changes) COL1 (3.57 fold changes)	Highly expressed: ALP (42.5 fold changes) COL1 (6.17 fold changes)	Lowest

2	Kamel et al. (2019) [64]	Alizarin red staining	Formation of mineralized nodules	Stronger formation of mineralized nodules	None	Expressed: ALP OCN	Highly expressed: ALP OCN	None

3	Sun et al. (2019) [44]	Alizarin red staining	Stronger formation of mineralized nodules	Formation of mineralized nodules	None	Highly expressed: ALP Runx2 OSX OCN (same)	Expressed: ALP Runx2 OSX (lowest) OCN	None
		ALP staining & ALP activity	Deeper staining in GMSCs compared with BMSCs/significantly higher activity	Less staining/active	None			

4	Xing et al. (2019) [61]	Alizarin red staining	Less formation of mineralized nodules	Stronger formation of mineralized nodules in PDLSCs followed by DPSCs	—	Expressed: ALP (lowest) Runx2 OSX (lowest) OCN COL1	Highly expressed: ALP, OSX (higher in PDL) Runx2 OCN, COL1 (higher in DP)	—

5	Angelopoulos et al. (2018) [56]	Alizarin red staining	Formation of mineralized nodules	Formation of mineralized nodules	None	—	—	—

6	Zhang et al. (2018) [57]	Alizarin red staining	Least formation of mineralized nodules in GMSCs and UCMSCs	Stronger formation of mineralized nodules in BMSCs and ADSCs followed by PDLSCs, DFSCs, and DPSCs	—	Expressed: ALP (same) COL1 (same) OCN (less)	Expressed: ALP COL1 OCN	—
		ALP staining & ALP activity	Less staining in GMSCs and UCMSCs	Deeper staining in BMSCs, ADSCs, PDLSCs, and DFSCs, followed by DPSCs	—			

7	Ghaderi et al. (2018) [53]	Alizarin red staining	Less formation of mineralized nodules	Stronger formation of mineralized nodules	None	Very lowly expressed: BMP COL1 BGLA	Highly expressed: BMP (733 fold changes) COL1 (282 fold changes)	None

8	Aboushady et al. (2018) [62]	Alizarin red staining	Moderate formation of mineralized nodules	Stronger formation of mineralized nodules in BMSCs and lower in SSMSCs	—	Moderately expressed: Runx2 MMP-13	Highly expressed in BMSCs and lower in SSMSCs: Runx2 MMP-13	—
9	Ansari et al. (2017) [54]	Alizarin red staining	Formation of mineralized nodules	Formation of mineralized nodules	—	Expressed: Runx2 OCN	Expressed: Runx2 OCN	—
		Xylenol orange staining	XO-positive labeling	XO-positive labeling	—			

10	Kaibuchi et al. (2017) [46]	Alizarin red staining	Less formation of mineralized nodules	Strongest formation of mineralized nodules in PDLSCs and PSCs followed by BMSCs and ADSCs	—	Lowly expressed: COL1A1 Moderately expressed: COL3A1	Highly expressed in BMSCs and PSCs: COL1A1 COL3A1	—
		H&E	Fail to form cell sheets	BMSCs, ADSCs, and PSCs generate cell sheets, while PDLSCs fail to form cell sheets	—			

11	Gao et al. (2014) [55]	Alizarin red staining	Formation of mineralized nodules	Similar formation of mineralized nodules in PDLSCs Both GMSCs and PDLSCs were higher than DSCs	—	Moderately expressed: ALP Runx2 OCN COL1	Highly expressed for PDLSCs and lower for DSCs: ALP Runx2 OCN COL1	—
		ALP activity	Moderate activity	Higher activity in PDLSCs and lower in DSCs	—			

12	Gay et al. (2014) [49]	Alizarin red staining	Formation of mineralized nodules	Formation of mineralized nodules	None	Expressed: Runx2	Highly expressed in PDLSCs and various degrees of expression in other sources: Runx2	—

13	Yang et al. (2013) [60]	Alizarin red staining	Formation of mineralized nodules	Stronger formation of mineralized nodules	None	Expressed: Runx2 OCN COL1	Highly expressed: Runx2 OCN COL1	None
		ALP staining & ALP activity	Active	Higher activity	None			

14	Moshaverinia et al. (2012) [45]	Alizarin red staining	Less formation of mineralized nodules	Stronger formation of mineralized nodules in BMSCs and PDLSCs	None	Expressed: Runx2 OCN	Expressed: Runx2 OCN	
*In vitro* studies confirmed the results by ectopic bone formation								

15	Zorin et al. (2014) [63]	H&E staining	Exhibited Osteoinductive features	Exhibited Osteoinductive features	Less new bone formation	—	—	—
		Immunohisto chemical staining	Intense reaction for OCN, OPN, ALP, and COL1	Intense reaction for OCN, OPN, ALP, and COL1	Weak reaction			
16	Moshaverinia et al. (2013) [65]	Xylenol orange staining	Less formation of mineralized nodules	Strong formation of mineralized nodules in BMSCs followed by PDLSCs	None	Highly expressed: Runx2 Bone sialoprotein OCN	Highly expressed: Runx2 Bone sialoprotein OCN	None
		ALP activity	Lowest activity	Significantly higher activity in BMSCs followed by PDLSCs	None			
		Micro-CT	Considerable bone volume fraction	Significantly larger bone volume fraction in BMSCs and considerable volume in PDLSCs	None			
		H&E staining	New bone formation	Significant new bone formation in BMSCs followed by PDLSCs	Scaffold remnants & CT			

17	Otabe et al. (2012) [50]	Alizarin red staining	Stronger formation of mineralized bone nodules	Strong formation of mineralized bone nodules	Less	Highly expressed: Runx2 OCN	Highly expressed: Runx2 OCN	Lowest
18	Mensing et al. (2011) [47]	Von Kossa staining	Formation of mineralized nodules	Formation of mineralized nodules	None	Chondrogenic markers only	Chondrogenic markers only	—

19	Fournier et al. (2010) [51]	Alizarin red staining	Strong formation of mineralized nodules	Strong formation of mineralized nodules	None	*—*	*—*	—
		H&E staining	Formation of the new bone matrix	Formation of the new bone matrix	None			
		ALP staining	Positive reactivity	Positive reactivity	None			

20	Tomar et al. (2010) [58]	Alizarin red staining	Strong formation of mineralized tissue	ND		Expressed: ALP COL1 OCN Cbfa1 Osterix	ND	
		H&E staining	New bone formation	New bone formation	None			
		Von Kossa staining	Highly mineralized tissue	Highly mineralized tissue	None			
		Immunostaining	Intense positive reaction for osteogenic marker OCN	Intense positive reaction for osteogenic marker OCN	None			

**Table 4:** Summary of findings for *in vivo* studies (methodology).

No.	Author and year of publication	Animal model			Experimental groups/number				Stem cells						Bone defect	
		Species/strain	Sex/age (w)	Weight	Source of interest GMSCs	Other sources	Positive control	Negative control	Source	Isolation method	Dose	Delivery method	Scaffold or carrier	Fate tracing	Site & diameter (mm)	Induction method
1	Al-Qadhi et al. (2020) [48]	New Zealand rabbit	M 24	2.5-3 kg	GMSCs/9	BMSCs/9	—	Cell-free scaffold /9	Rabbit	Enzymatic	1 × 10^6^	Local	NanoBone	PKH26	Tibia/6.0	Surgical
2	Sun et al. (2019) [67]	C57BL/6J mice	NM 8	21.08 g	GMSCs/18	—	—	Cell-free medium/18	Human	Enzymatic	1 × 10^6^	Systematic	*α*-MEM	GFP	Maxilla/NM	Disease induced by ligation
3	Xu et al. (2014) [68]	C57BL/6J mice	M 7	NM	GMSCs/18	—	—	Cell-free medium/18	Human	Enzymatic	1 × 10^6^	Systematic	*α*-MEM	GFP	Mandible/1.5	Surgical
4	Moshaverinia et al. (2014) [66]	Beige nude mice	NM 20	NM	GMSCs/4	PDLSCs/4	BMSCs/4	Cell-free scaffold/4	Human	Enzymatic	4 × 10^6^	Local	RGD-modified alginate	—	Calvaria/0.5	Surgical
5	Yu et al. (2012) [69]	Beagle dogs	M 4	10-11 kg	GMSCs/4	—	—	Cell-free scaffold/contralateral side	Human	Enzymatic	1 × 10^4^	Local	Cell sheet	GFP	Mandible/5.0	Surgical
6	Wang et al. (2011) [52]	SD rats	F 6-8	160-180 g	GMSCs/5	—	—	Cell-free matrix/5	Human	Enzymatic	1‐2 × 10^6^	Local	Type 1 collagen gel	GFP	Mandible/1.0	Surgical
					GMSCs/3			Cell-free matrix/contralateral side							Calvaria/5.0	

**Table 5:** Summary of findings for the *in vivo* studies (primary outcome result: new bone formation).

No.	Author and year of publication	Morphological appearance	Functional verification	Phenotypic verification GMSCs	Last time point/weeks	Method of analysis	GMSCs	Other sources	Positive control	Negative control (without stem cells)	Statistical analysis
1	Al-Qadhi et al. (2020) [48]	Fibroblast spindle-like cells	ND	✓: CD105, CD90, CD73, CD29 ✖: CD34, CD45	6	H&E staining	New bone formed at the defect borders	New bone formed at the defect border and at the center, bridging the defect	—	Newly formed bone restricted to lateral walls	Significant difference between each stem cell group and the control while nonsignificant between 2 MSC groups
						MT staining	Abundance of red color indicating lamellated bone	Abundance of red color indicating lamellated bone	—	Intermingling areas of woven bone and lamellated bone	Significant between each stem cell group and the control while nonsignificant between 2 MSC groups

2	Sun et al. (2019) [67]	Fibroblast spindle-like cells	Osteogenic Adipogenic	✓: CD105, CD90, CD73 ✖: CD45	4	H&E staining	More newly formed bone and higher alveolar bone heights	—	—	Limited newly formed bone	Significant decrease in alveolar bone loss in the stem cell group compared to the control
						MT staining	Abundance of red color indicating more mature bone	—	—	Less mature bone	

3	Xu et al. (2014) [68]	Fibroblast spindle-like cells	Osteogenic Adipogenic Chondrogenic	✓: CD105, CD73, CD29, CD44, STRO-1 ✖: CD144, CD31, HLA-DR, CD34, CD45	3	H&E staining MT^#^	More newly formed bone than the control group			Limited newly formed bone	Significant difference between the stem cell and control groups

4	Moshaverinia et al. (2014) [66]	Fibroblast-like cells	Osteogenic	✓: CD146, CD166 ✖: CD34	8	H&E staining	Low amount of new bone	High amount of new bone	High amount of new bone	Less newly formed bone	Significant difference between dental stem cell sources and the control
						MT staining	Viable mature woven bone formation with a lamellate pattern	Viable mature woven bone formation with a lamellate pattern	Viable mature woven bone formation with a lamellate pattern	Bundles of collagenous fibers and unresorbed scaffolds	
						Immunohistochemical staining	Mild expression of Runx2 and OCN	Strong expression of Runx2 and OCN	Strong expression of Runx2 and OCN	No expression	
						Micro-CT	Lower newly formed bone in comparison to BMSCs & PDLSCs	—	Largest amount of bone	No bone regeneration	Significant difference between dental stem cell sources and the control
5	Yu et al. (2012) [69]	Fibroblast-like cells	Osteogenic Adipogenic	✓: CD105, CD90, CD73, CD44, CD29, STRO-1 ✖: CD14, CD45, CD144, CD31, HLA-DR	8	H&E staining	More newly formed bone	—	—	Less newly formed bone	Significant difference between the stem cell and control groups
						Picrosirius red staining	No mineralization but newly formed Sharpey's fibers	—	—	No mineralization but newly formed Sharpey's fibers	—

6	Wang et al. (2011) [52]	Fibroblast-like cells	Osteogenic Adipogenic Chondrogenic	✓: CD105, CD90, CD29, STRO-1 ✖: CD34, CD45	8	H&E staining	Newly formed bone with the well-mineralized trabecular structure	—	—	Abundant connective tissue and very limited newly formed bone	NR
						Immunohistochemical staining	Strong expression of GFP, OPN, and COL1	—	—	Weak expression of GFP, OPN, and COL1	NR

## Supplementary Materials

The current research has the following supplementary materials:

Supplementary 1 Supplementary material I. PRISMA checklist.

Supplementary 2 Supplementary material II. Detailed search strategy.

Supplementary 3 Supplementary material III. List of excluded studies.

Supplementary 4 Supplementary material IV. Detailed *in vitro* assessment.
