# Supplementary material for: The Gingiva from the Tissue Surrounding the Bone to the Tissue Regenerating the Bone: A Systematic Review of the Osteogenic Capacity of Gingival Mesenchymal Stem Cells in Preclinical Studies
Source: Stem Cells Int. 2021 Jun 12;2021:6698100. doi: 10.1155/2021/6698100 (PMC8218920; doi:10.1155/2021/6698100)
Supplement: Supplementary 2 — Supplementary material II. Detailed search strategy. [file 6698100.f2.pdf]

Supplementary Material II: search strategy.

Database/ Date of search	Search query	hints
PubMed 22/6/2020 16:05	((bone defect OR bone defects OR bone degradation OR bone disease OR bone disorder OR bone loss OR bone deformation OR bone destruction OR bone injury OR bone fracture) **OR** (animal model OR animal models OR experimental animal OR experimental animals OR 'laboratory animal OR 'laboratory animals OR in vivo study OR in vivo study OR in vitro study OR in vitro OR 'in vitro studies OR in vitro technique OR in vitro techniques OR cell culture technique OR cell culture method OR cell culture techniques OR culture technique OR culture techniques OR preclinical study)) **AND** (gingival mesenchymal stem cell OR gingival mesenchymal stem cells OR gingiva derived mesenchymal stem cell OR gingival tissue derived mesenchymal stem cells OR gingiva derived stromal cell OR gingiva derived stromal cells OR multipotent gingival stromal cell OR multipotent gingiva stromal cells OR multipotent gingiva progenitor cells OR gingiva stem cells)	374
Embase using quick search* 20/6/2020 5:00 AM	(**'bone defect'**:ti,ab,kw OR **'bone defects'**:ti,ab,kw OR **'bone degradation'**:ti,ab,kw OR **'bone disease'**:ti,ab,kw OR **'bone disorder'**:ti,ab,kw OR **'bone loss'**:ti,ab,kw OR **'bone deformation'**:ti,ab,kw OR **'bone destruction'**:ti,ab,kw OR **'bone injury'**:ti,ab,kw OR **'bone fracture'**:ti,ab,kw OR **'animal model'**:ti,ab,kw OR **'animal models'**:ti,ab,kw OR **'experimental animal'**:ti,ab,kw OR **'experimental animals'**:ti,ab,kw OR **'laboratory animal'**:ti,ab,kw OR **'laboratory animals'**:ti,ab,kw OR **'in vivo study'**:ti,ab,kw OR **'in vitro study'**:ti,ab,kw OR **'in vitro'**:ti,ab,kw OR **'in vitro studies'**:ti,ab,kw OR **'in vitro technique'**:ti,ab,kw OR **'in vitro techniques'**:ti,ab,kw OR **'cell culture technique'**:ti,ab,kw OR **'cell culture method'**:ti,ab,kw OR **'cell culture techniques'**:ti,ab,kw OR **'culture technique'**:ti,ab,kw OR **'culture techniques'**:ti,ab,kw OR **'preclinical study'**:ti,ab,kw) AND (**'gingival mesenchymal stem cell'**:ti,ab,kw OR **'gingival mesenchymal stem cells'**:ti,ab,kw OR **'gingiva derived mesenchymal stem cell'**:ti,ab,kw OR **'gingival tissue derived mesenchymal stem cells'**:ti,ab,kw OR **'gingiva derived stromal cell'**:ti,ab,kw OR **'gingiva derived stromal cells'**:ti,ab,kw OR **'multipotent gingival stromal cell'**:ti,ab,kw OR **'multipotent gingiva stromal cells'**:ti,ab,kw OR **'multipotent gingiva progenitor cells'**:ti,ab,kw OR **'gingiva stem cells'**:ti,ab,kw)	72
Embase using PICO 20/6/2020 5:00 AM	(**'bone defect'**/exp OR **'bone defect'** OR **'bone degradation'** OR **'degradation, bone'** OR **'bony defect'** OR **'bone defects'** OR **'bone disease'**/exp OR **'bone disease'** OR **'bone diseases'** OR **'bone disorder'** OR **'disease, bone'** OR **'bone**	16

	**loss'** OR **'bone deformation'**/exp OR **'bone destruction'**/exp OR **'bone destruction'** OR **'bone injury'**/exp OR **'bone injury'** OR **'bone fracture'**) AND (**'gingival mesenchymal stem cell'**/exp OR **'gingival mesenchymal stem cell'** OR **'gingival mesenchymal stem cells'** OR **'gingiva derived mesenchymal stem cell'**/exp OR **'gingiva derived mesenchymal stem cell'** OR **'gingival tissue derived mesenchymal stem cells'** OR **'gingiva derived stromal cell'** OR **'gingiva derived stromal cells'** OR **'multipotent gingival stromal cell'** OR **'multipotent gingiva stromal cells'** OR **'multipotent gingiva progenitor cells'** OR **'gingiva stem cells'**) AND (**'animal model'**/exp OR **'animal model'** OR **'animal models'**/exp OR **'animal models'** OR **'experimental animal'**/exp OR **'animal, laboratory'** OR **'animals, laboratory'** OR **'experimental animal'** OR **'experimental animals'** OR **'laboratory animal'** OR **'laboratory animals'** OR **'in vivo study'**/exp OR **'in vivo study'** OR **'in vitro study'**/exp OR **'in vitro'** OR **'in vitro studies'** OR **'in vitro study'** OR **'in vitro technique'** OR **'in vitro techniques'** OR **'cell culture technique'**/exp OR **'cell culture method'** OR **'cell culture technique'** OR **'cell culture techniques'** OR **'culture technique'** OR **'culture techniques'** OR **'preclinical study'**/exp OR **'preclinical study'**)	
Web of science 24/6/2020 4:02 AM	TI=(bone defect* OR bone defects* OR bone degradation* OR bony defect* OR bone disease* OR bone disorder* OR bone loss* OR bone deformation* OR bone destruction* OR bone injury* OR bone fracture) **OR** TI=(animal model* OR animal models* OR experimental animal* OR experimental animals* OR laboratory animal* OR laboratory animals* OR in vivo study* OR in vivo study* OR in vitro study* OR in vitro* OR in vitro studies* OR in vitro technique* OR in vitro techniques* OR cell culture method* OR cell culture technique* OR cell culture techniques* OR culture technique* OR culture techniques* OR preclinical study) **AND** TI=(gingival mesenchymal stem cell* OR gingival mesenchymal stem cells* OR gingiva derived mesenchymal stem cell* OR gingiva derived mesenchymal stem cells* OR gingival tissue derived mesenchymal stem cells* OR gingiva derived stromal cell* OR gingiva derived stromal cells* OR multipotent gingiva stromal cell* OR 'multipotent gingiva progenitor cells* OR gingiva stem cells)	168
Scopus 23/6/2020 12:00 PM	((bone defect OR bone defects OR bone degradation OR bone disease OR bone disorder OR bone loss OR bone deformation OR bone destruction OR bone injury OR bone fracture) **AND** (gingival mesenchymal stem cell OR gingival mesenchymal stem cells OR gingiva derived mesenchymal stem cell OR gingival tissue derived mesenchymal stem cells OR gingiva derived stromal cell OR gingiva	24

	derived stromal cells OR multipotent gingival stromal cell OR multipotent gingiva stromal cells OR multipotent gingiva progenitor cells OR gingiva stem cells)) **AND** (animal model OR animal models OR experimental animal OR experimental animals OR 'laboratory animal OR 'laboratory animals OR in vivo study OR in vivo study OR in vitro study OR in vitro OR 'in vitro studies OR in vitro technique OR in vitro techniques OR cell culture technique OR cell culture method OR cell culture techniques OR culture technique OR culture techniques OR preclinical study)	

Embase search using Pico field resulted in a few retrieved articles so we used quick search instead.
