# Supplementary material for: The Gingiva from the Tissue Surrounding the Bone to the Tissue Regenerating the Bone: A Systematic Review of the Osteogenic Capacity of Gingival Mesenchymal Stem Cells in Preclinical Studies
Source: Stem Cells Int. 2021 Jun 12;2021:6698100. doi: 10.1155/2021/6698100 (PMC8218920; doi:10.1155/2021/6698100)
Supplement: Supplementary 3 — Supplementary material III. List of excluded studies. [file 6698100.f3.pdf]

Supplementary Material III: Reasons for exclusion during phase 2: full-text article screen.

No.	Author and year of publication	Title	Reason for exclusion
1.	Nugraha, et al., 2018	Gingival Mesenchymal Stem Cells from Wistar Rat's Gingiva (Rattus Novergicus)–Isolation and Characterization (In Vitro Study)	No MSCs comparator group/ gingival fibroblast
2.	Bennur et al., 2020	Gold nanoparticles biosynthesized by Nocardiopsis dassonvillei NCIM 5124 enhance osteogenesis in gingival mesenchymal stem cells. Appl. Microbiol. Biotechnol. 104, 4081–4092. https://doi.org/10.1007/s00253-020-10508-z	No MSCs comparator group/ cells free-scaffold
3.	Coimbra et al., 2015	Clopidogrel enhances mesenchymal stem cell proliferation following periodontitis.	Irrelevant main intervention/ BMSCs
4.	Diniz et al., 2016	Gingival Mesenchymal Stem Cell (GMSC) Delivery System Based on RGD-Coupled Alginate Hydrogel with Antimicrobial Properties: A Novel Treatment Modality for Peri-Implantitis.	Irrelevant outcome
5.	Proksch et al., 2019	Comparative differentiation analysis of distinct oral tissue-derived cells in response to osteogenic stimulation.	Mixed cells, not pure MSCs
6.	Santamaría et al., 2017	Comparison of periodontal ligament and gingiva-derived mesenchymal stem cells for regenerative therapies	PDLSCs from diseased tissue/ not healthy tissue
7.	Sadrabadi et al., 2020	An engineered cell-laden adhesive hydrogel promotes craniofacial bone tissue regeneration in rats	No MSCs comparator/different composite of scaffold
8.	Treves-Manusevitz et al., 2013	Stem cells of the lamina propria of human oral mucosa and gingiva develop into mineralized tissues in vivo.	Mixed cells in one construct
9.	Wu et al., 2020	CD39 Produced from Human GMSCs Regulates the Balance of Osteoclasts and Osteoblasts through the Wnt/β-Catenin Pathway in Osteoporosis	No MSCs comparator/dermal fibroblast
