# Supplementary material for: The Gingiva from the Tissue Surrounding the Bone to the Tissue Regenerating the Bone: A Systematic Review of the Osteogenic Capacity of Gingival Mesenchymal Stem Cells in Preclinical Studies
Source: Stem Cells Int. 2021 Jun 12;2021:6698100. doi: 10.1155/2021/6698100 (PMC8218920; doi:10.1155/2021/6698100)
Supplement: Supplementary 4 — Supplementary material IV. Detailed in vitro assessment. [file 6698100.f4.pdf]

Table: Quality assessment of in vitro studies depending on the Guidelines for Reporting Pre-clinical In Vitro Studies on Dental Materials which was based on the modification of CONSORT checklist.

Item	Abedian et al., 2020	Kamel et al., 2019	Sun et al., 2019	Xing et al., 2019	Angelopouls et al., 2018	Zhang et al., 2018	Ghaderi et al., 2018	Aboushady et al., 2018	Ansari et al., 2017	Kaibuchi et al., 2017	Gao et al., 2014	Gay et al., 2013	Yang et al., 2013	Moshaverinia et al., 2012	Zorin et al., 2014	Moshaverinia et al., 2013	Otabe et al., 2012	Mensing et al., 2011	Fournier et al., 2010	Tomar et al., 2010
1	Yes	Yes	Yes	Yes	Yes	Yes	Yes	Yes	Yes	Yes	Yes	Yes	Yes	Yes	Yes	Yes	Yes	Yes	Yes	Yes
2a	Yes#	Yes	Yes	Yes	Yes	Yes	Yes	Yes#	Yes	Yes	Yes#	Yes	Yes	Yes	Yes	Yes	Yes	Yes	Yes#	Yes#
2b	Yes	Yes	Yes	Yes	Yes	Yes	Yes#	Yes	Yes	Yes	Yes	Yes	Yes	Yes	Yes	Yes	Yes	Yes	Yes#	Yes
3	Yes	Yes	Yes	Yes	Yes	Yes	Yes	Yes	Yes	Yes	Yes	Yes	Yes	Yes	Yes	Yes	Yes	Yes	Yes	Yes
4	Yes	Yes	Yes	Yes	Yes	Yes	Yes	Yes	No	No	Yes	Yes	Yes	Yes	No	Yes	Yes	Yes	Yes	Yes
5	No	No	No	No	No	No	No	No	No	No	No	No	No	No	No	No	No	No	No	No
6	No	No	No	No	No	Yes#	No	No	No	No	No	No	No	No	No	No	No	No	No	No
7	No	No	No	Yes#	No	Yes#	No	No	No	No	No	No	Yes#	No	No	No	No	No	No	No
8	No	No	No	No	No	No	No	No	No	No	No	No	No	No	No	No	No	No	No	No
9	No	No	No	No	No	No	No	No	No	No	No	No	No	No	No	No	No	No	No	No
10	Yes	Yes	Yes	Yes	Yes	Yes	Yes#	Yes	Yes	Yes	Yes	Yes	Yes	Yes	Yes	Yes	Yes	Yes	Yes*	Yes*
11	Yes	Yes	Yes	Yes	Yes	Yes	Yes#	Yes	Yes#	Yes	Yes	Yes	Yes	Yes	Yes	Yes	Yes	Yes	Yes	Yes
12	No	No	Yes+	No	No	Yes	No	No	Yes	Yes	No	No	Yes	Yes	Yes	Yes	Yes	Yes	No	No
13	Yes	Yes°	Yes	Yes	Yes	Yes	No	No	Yes	Yes	No	Yes	Yes	Yes	Yes	Yes	Yes	Yes	Yes	Yes
14	No	Yes°	Yes°	Yes°	Yes°	Yes°	Yes°	Yes°	Yes°	Yes°	Yes°	Yes°	Yes°	Yes°	Yes°	Yes°	Yes°	Yes°	Yes°	Yes°

Yes#; reported but not enough, Yes; reported but not presented well, Yes°; protocol was approved by local authority without web registration.*
